# A local insult of okadaic acid in wild-type mice induces tau phosphorylation and protein aggregation in anatomically distinct brain regions

**DOI:** 10.1186/s40478-016-0300-0

**Published:** 2016-03-31

**Authors:** Siân Baker, Jürgen Götz

**Affiliations:** Clem Jones Centre for Ageing Dementia Research (CJCADR), Queensland Brain Institute (QBI), The University of Queensland, St Lucia Campus, Brisbane, QLD 4072 Australia

**Keywords:** Alzheimer’s disease, Okadaic acid, Phosphorylation, Protein phosphatase 2A, Spreading, Tau, Tauopathy

## Abstract

In Alzheimer’s disease (AD), the distribution and density of neurofibrillary tangles, a histological hallmark comprised predominately of phosphorylated tau protein, follows a distinct pattern through anatomically connected brain regions. Studies in transgenic mice engineered to regionally confine tau expression have suggested spreading of tau within neural networks. Furthermore, injection of protein lysates isolated from brains of transgenic mice or patients with tauopathies, including AD, were shown to behave like seeds, accelerating tau pathology and tangle formation in predisposed mice. However, it remains unclear how the initiation of primary aggregation events occurs and what triggers further dissemination throughout the neural system. To consolidate these findings, we pursued an alternative approach to assess the spreading of endogenous phosphorylated tau. To generate endogenous seeds, 130 nl of 100 μM protein phosphatase 2A inhibitor okadaic acid (OA) was injected unilaterally into the amygdala of 8-month-old C57Bl/6 wild-type mice. OA was detected in brain tissue by ELISA, and found to be restricted to the injected hemispheric quadrant, where it remained detectable a week post-injection. OA injection induced tau phosphorylation that was observed not only at the injection site but also in anatomically distinct areas across both hemispheres, including the cortex and hippocampus 24 h post-injection. An increase in insoluble tau was also observed in both hemispheres of injected brains by 7 days. Furthermore, thioflavin-S detected protein aggregation at the injection site and in the cortex of both injected and contralateral hemispheres. OA injection induced no thioflavin-positivity in tau knock-out mice. The data demonstrates that a local OA insult can rapidly initiate changes in protein phosphorylation, solubility and aggregation at anatomically distant sites. This model suggests that tau phosphorylation can be both a primary response to an insult, and a secondary response communicated to non-exposed brains regions. The study highlights the use of OA to assist in understanding the initiation of tau spreading in vivo.

**Electronic supplementary material:**

The online version of this article (doi:10.1186/s40478-016-0300-0) contains supplementary material, which is available to authorized users.

## Introduction

Alzheimer’s Disease (AD) is characterised by the presence of two types of protein aggregates, amyloid-β (Aβ)-containing plaques and neurofibrillary tangles (NFTs), the latter containing the microtubule-associated protein tau in a hyperphosphorylated form [1]. Regarding the latter, their distribution and density follows a distinct pattern through anatomically connected brain regions. NFTs constitute the basis for the Braak staging in AD [2] that is routinely used for post-mortem diagnosis [3]. This staging has led to the suggestion of a stereotypical spread of tau pathology, proposed to be a result of cross-synaptic spreading of a corruptive pathological protein species from a diseased to a healthy neuron in a “prion-like” manner along neuronal connections, and has been termed the spreading hypothesis [4, 5]. In Prion disease it has been demonstrated that a pathological protein seed is capable of inducing misfolding of its native form and propagation of pathology between neurons, with severe down-stream functional consequences [6]. In recent years, by extrapolation, cell-to-cell protein spreading and seeding have been suggested to underlie multiple neurodegenerative diseases [7, 8] including AD, with a focus on a histological assessment and so far not so much on ensuing clinical changes [9].

To address tau spreading in animal models, transgenic mice have been used that express mutant forms of tau found in familial cases of frontotemporal dementia (FTD), using promoter elements that confer broad expression of the transgene [10]. Two principal approaches have been pursued. Firstly, by using inducible models and brain area-specific promoters to restrict the expression of FTD mutant tau to brain regions such as the entorhinal cortex, an early site of tau pathology in human AD, pathologically folded tau was found in neighbouring regions, indicative of tau spreading [11, 12]. Although complete restriction of the transgene has been refuted in a subsequent study [13], it has been argued that the changes seen in these neighbouring regions cannot be traced back to the low degree of ‘transgene leakage’. In a second approach, injection of mouse brain lysates isolated from FTD tau mutant mice into pre-symptomatic recipient mice has been shown to cause an accelerated, mature tau pathology both at the injection site and also, in neighbouring regions [14]. Moreover, following the injection of the corresponding brain extracts, the hallmark lesions of tauopathies such as AGD, PSP and CBD have been recapitulated [15]. While there has been indication that endogenous tau is not required to initiate tau pathology, recent data has demonstrated that a tau knock-out background does not affect the ability of tau to propagate but can reduce the pathological alterations induced by the ‘seed’ [16]. Furthermore, injection of synthetic tau fibrils alone are able to induce the propogation of tau pathology [17, 18]. However, the use of mature fibrillar tau seeds in previous seeding studies supersedes the intial seeding event causal of the primary aggregation events in the human disease state. Therefore, whether the effects observed in these studies are reliant on the introduction of a preformed mature tau seeds remains unknown.

To address this question we used a novel approach by creating a localised insult through injection of a single low dose of the protein phosphatase inhibitor okadaic acid (OA) in a very small volume, unilaterally targeting the amygdala, a limbic area affected early on in AD [19]. Although OA predominantly inhibits PP1 and PP2A, it has a much higher affinity for the latter [20]. OA inhibits the ability of PP2A to dephosphorylate tau resulting in tau hyperphosphorylation, which is exacerbated by an increase in kinase activity [21]. Increases of endogenous PP2A inhibitors have been reported in human cases of AD, where they co-localise with phospho-tau and early NFT markers [22]. Therefore, we propose that targeting the inhibition of PP2A will provide a spreading stimulus which precedes mature filament formation. Previous animal studies have primarily utilised OA at high doses or by chronic delivery to the hippocampus or ventricular system to recapitulate multiple features of both biochemical and behavioural dysfunctions of AD [23–25], whereas we used a single low dose of 10 ng. Secondly, through using a low 130 nl volume of 100 μM OA we aimed to avoid spill-over, to ensure specific OA exposure. Finally, by using wild-type mice we were able to investigate the potential for endogenous tau phosphorylation to initiate spreading in the absence of tau mutations or external preformed tau seeds, to address whether these effects can be observed in brain regions anatomically distinct to that of the primary insult.

## Materials and methods

### Experimental design

The objective of this study was to investigate consequences of localised OA-induced PP2A inhibition on tau phosphorylation at both the injection site and across the brain. We injected 8 month-old female C57Bl/6 mice with a single low dose of 10 ng OA to the lateral amygdala. By targeting the lateral amygdala we are able to use areas previously reported as susceptible to tau seeding as secondary spreading targets, such as of the hippocampus. The co-ordinates were chosen in order to avoid passing through the ventricle or the hippocampal formation, and the lateral amygdala was chosen due to its confinement within the divergence of the external capsule, to aid in the restriction of OA diffusion. DMSO was used to resuspend and solubilise OA and was volume-matched as a control throughout. An OA-competitive ELISA was used to confirm injection and define diffusion from the injection site. At 24 h and 7 d post-injection, tau phosphorylation was quantified at the injection site and at anatomically distinct regions of interest throughout the brain using immunohistochemistry for the tau phospho-epitope AT180 (pTh231/pSer235) [26]. Transition of tau into an insoluble state was quantified by Western blotting of lysates fractionated by solubility. Aggregation was assessed by reactivity with thioflavin-S that detects β-pleated sheet structures [27].

### Animals

Female wild-type C57BL/6 mice were used from 6 to 8 months of age. Female *MAPT* KO mice [28] were used as controls from 3 to 8 months of age. All experiments were carried out in compliance with ethical approval from the UQ animal welfare unit (QBI/412/14/NHMRC; QBI/027/12/NHMRC).

### Stereotaxic surgery

Mice were anaesthetised with isoflurane vaporised in oxygen. The head was shaved and depilated before being placed securely in ear bars. Mice were injected unilaterally with 130 nl of 100 μM OA (Sigma) solubilised in DMSO or DMSO alone to the lateral amygdala (Bregma coordinates: anterior/posterior −1.94, medial/lateral −3.15, dorsal/ventral −4.5) at an infusion rate of 60 nl/min. The same volume of the dye Evans Blue was injected in the same manner to confirm the injection site. Following the injection, the needle was left in place for 5 min before being slowly withdrawn, and the scalp was securely sutured. Once reflexes returned, postoperative analgesia was administered subcutaneously (1 mg/kg torbugesic) and mice were returned to their home cages. Mice were left to recover for 30 min, 24 h or 7 d. For histology, mice were anaesthetised with a lethal dose of pentabarbitone before transcardial perfusion with 30 ml PBS followed by 30 ml 4 % paraformaldehyde. Brains were removed from the skull and post-fixed overnight at 4 °C. For protein extraction, mice were perfused with PBS alone before being snap-frozen in liquid nitrogen. For the OA ELISA, brains were snap-frozen without perfusion.

### Histological tissue preparation and immunocytochemistry

Brains were dissected into forebrain, hindbrain and cerebellum before processing by paraffin embedding as described [29]. Sections were analysed by immunohistochemistry using the phospho-specific antibody AT180 (Thermo Fisher) as described [30].

### Thioflavin-S staining

Slides were dewaxed and rehydrated, followed by a 1 min incubation in 70 % ethanol and a subsequent 1 min incubation in 80 % ethanol. Slides were incubated in filtered 1 % thioflavin-S (Sigma) in 80 % ethanol for 15 min at room temperature (RT), protected from light. Slides were washed in 80 % ethanol, 70 % ethanol and twice in milliQ water for 1 min each, before incubation in ice-cold high phosphate buffer (411 mM NaCl, 8.1 mM KCl, 30 mM NA_2_HPO_4_, 5.2 mM KH_2_PO_4_, pH7.2) for 15 min at RT, protected from light. Slides were washed twice for 1 min in milliQ water before mounting in 50 % glycerol sealed with nail varnish and stored at 4 °C in the dark.

### Neuropathological quantification

Stained sections (3 per condition) were imaged using the slide scanner (Zeiss) at 20 × magnification and cropped into individual sections for analysis using the ImageJ software. Designated regions of interest (ROIs) were drawn for each brain region and phospho-tau immunoreactivity was quantified as a percentage area. The threshold for positive labelling was determined using the normal distribution of immunoreactivity from the control group and set at two standard deviations from the mean.

### Protein fractionation and western blotting

Samples were kept on dry ice and weighed individually. Proteins were extracted sequentially based on insolubility as previously described [31, 32]. Breifly, brain tissue was homogenised in 6 × volume of RAB buffer (0.1 M MES pH7.2, 1 mM EGTA, 0.5 mM MgSO4, 0. 75 M NaCl, plus phosphatase/protease inhibitors) in the TissueLyserLT (Qiagen) for 6 min at maximum speed. The homogenate was spun at 21,000 g for 90 min at 4 °C and the supernatant collectedand stored at −80 °C as the RAB soluble fraction. The remaining pellet was homogenised in the same volume of RIPA buffer to extract remaining insoluble proteins (10 × from Cell Signalling, plus phosphatase/protease inhibitors) and spun at 21,000 g for 90 min at 4 °C. The supernatant was collected and stored at −80 °C as the RIPA soluble fraction. Protein concentration in samples was determined by a BCA assay (Pierce) and 30 μg of protein was run per sample on a 10 % gel. Proteins were transferred onto Immobilon-FL membrane (Millipore) using the Trans-blot Turbo (BioRad) and blocked in Odyssey blocking buffer for 1 h at RT before incubation overnight in primary antibody (total tau (DAKO) 1:1000, AT180 1:500, AT8 1:500, AT270 1:1000, nitrated tau 1:100 (Thermofisher), ser422 1:1000 (GeneTex), ser262 1:1000 (ProSci), s235 1:1000 (Novus biologicals)). The next day membranes were washed in TBS-Tween (0.05 %) and incubated in secondary fluorescent antibodies (Licor, 1:20,000) for 30 min. Membranes were further washed in TBS-Tween before being visualised using the Odyssey Fc (Licor). Protein levels calculated using image studio software (Licor) and were normalised to actin.

### Competitive okadaic acid ELISA

The OA ELISA was performed by adapting a protocol for the detection of OA in mussels and salt water samples, using a competitive ELISA kit from Bioo Scientific. Briefly, frozen tissue was homogenised without buffer in the TissueLyserLT for 6 min at maximum speed. 50 % methanol was added at 1 ml/0.25 g of tissue. Samples were vortexed for 5 min, then centrifuged at 4000 rpm for 10 min. The supernatant was transferred to a new tube and heated at 75 °C for 5 min before a further 4000 rpm, 10 min centrifugation. The supernatant was collected and diluted 1:1 with 1 × extraction buffer/methanol (provided by the kit). 50 μl of OA standard or 50 μl of sample were added in duplicate to the provided secondary antibody-coated 96 well-plate. Subsequently, 50 μl of OA-HRP conjugate followed by 50 μl anti-OA antibody were added to each well, immediately followed by mixing by pipetting up and down once. The plate was manually rocked for one min and the sample incubated at RT in the dark for 30 min. The plate was washed 3 × with 200 μl of the provided wash solution. After the last wash the plate was inverted and gently tapped dry on paper towels. 100 μl TMB substrate was added, the plate manually rocked for one min and then incubated at RT in the dark for 15 min. 100 μl of stop buffer were added to stop the enzyme reaction and the plate was read on the POLARstar Optima (BMG Labtech) at 450 nm wavelength.

### Statistical analysis

Results are displayed as mean with error bars representing ± the standard error of the mean. Statistical analyses were conducted using the student’s unpaired t-tests or one way ANOVA test with appropriate post-hoc analyses for multiple comparisons. GraphPad Prism was used to perform statistical tests with statistical significance set to a *P* < 0.05.

## Results

### Okadaic acid (OA) stereotaxically injected into wild-type brains is restricted to the site of injection

**Fig. 1:**
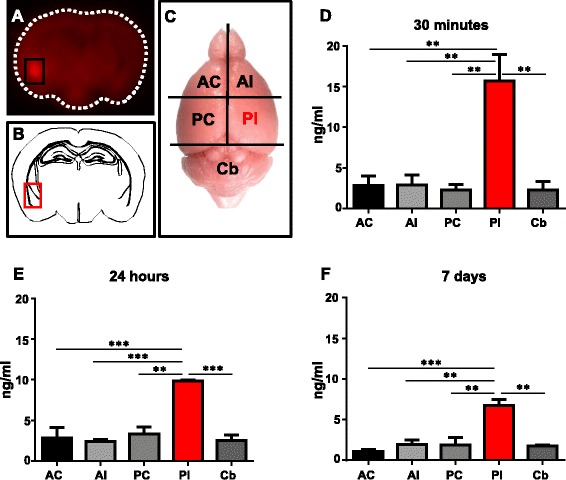
Brain dissection to confirm injection site. **a** Mice are unilaterally injected at the co-ordinates for the lateral amygdala with Evans blue dye and culled 30 min later. 1 mm thick brain sections are imaged at 700 nm where injected dye fluoresces red. **b** Comparison to neuroanatomy at Bregma −1.94 mm confirms injection location to the lateral amygdala. **c** Mice brains are dissected into the anterior contralateral (AC), anterior ipsilateral (AI), posterior contralateral (PC), posterior ipsilateral (PI) and cerebellum (Cb) for analysis by ELISA and western blotting. The PI fraction contains the lateral amygdala injection site. **d**-**f** OA concentration in brain tissue is quantified by ELISA. Significant levels of OA are detected in the PI fraction alone at all time points, with no significant difference observed between any of the non-injected fractions, with concentrations comparable to homogenised tissue controls (Additional file 1: Figure S1). The PI fraction maintains significantly elevated levels of OA at 24 h and 7 days after injection, although the concentration is reduced. (*n* = 3, **p < 0.01, ***p < 0.001)

To establish the OA injection paradigm in wild-type mice, the dye Evans Blue was stereotaxically injected into the lateral amygdala, a site with prominent tau pathology in both AD and in tau transgenic mouse models [33]. The mice were sacrificed 30 min post-injection, immersion fixed and dissected into 1 mm sections. Visualization using a 700 nm laser revealed fluorescence confined to the injection site (Fig. 1a), as validated by reference to the Allen mouse brain atlas (Fig. 1b). This analysis further demonstrated that the volume and pressure settings used did not cause passing of the dye into the ventricular system, although it is possible that OA has different diffusion properties to Evans blue.

To specifically investigate whether over time, OA would diffuse away from the injection site into adjacent brain tissue, we followed injection of OA with a commercially available competitive ELISA. Mice were sacrificed 30 min, 24 h and 7 days post-injection, respectively, and brains were dissected into four quadrants and the cerebellum, with the posterior ipsilateral (PI) quadrant containing the OA injection site (Fig. 1c). This allowed quantifiable detection of OA in brain tissue for the first time (Fig. 1d-f). OA was detectable at significantly elevated levels in the PI fraction at all time points investigated, although its concentration was reduced over time. It is unlikely that the observed reduction in the PI quadrant over time is due to diffusion of OA into the other quadrants or cerebellum as no significant changes in signal were observed in tissue from non-injected regions. Due to the novelty of this approach we cannot rule out sub-detectable levels of OA in the non-injected hemisphere, however, the levels observed in non-injected tissue were comparable to the background observed in non-injected brain tissue controls (Additional file 1: Figure S2B). Therefore, this indicates that by keeping the OA dose and volume low, and by choosing co-ordinates that avoid the ventricular system, significant spreading of the inhibitor from the injection site was restricted, minimising OA exposure in non-target regions.

### Okadaic acid induces tau phosphorylation in distinct brain regions across both hemispheres

**Fig. 2:**
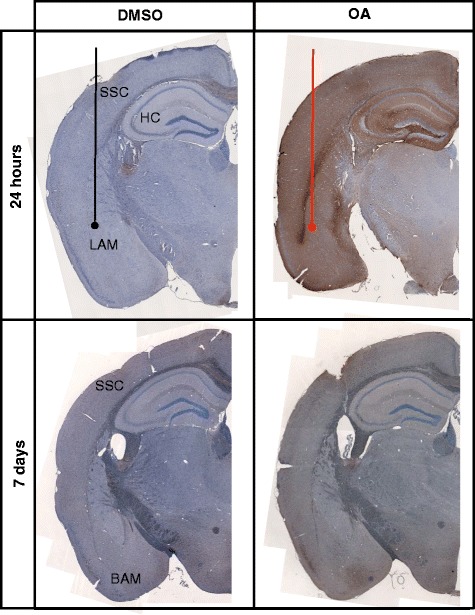
Characterisation of tau pathology. Representative coronal sections from the injection site are stained for phospho-tau with AT180 and counterstained with haematoxylin for cell bodies. Tissue injected with DMSO have relatively low levels of phospho-tau immunoreactivity. At 24 h phospho-tau is abundant at the injection site in the lateral amygdala (LAM) and can be seen throughout the layers of the cortex (CX) and hippocampus (HC) in OA injected. At 7 days phospho-tau persists primarily in the basal amygdala (BAM) and somatosensory cortex (SSC) in the OA injected mice

**Fig. 3:**
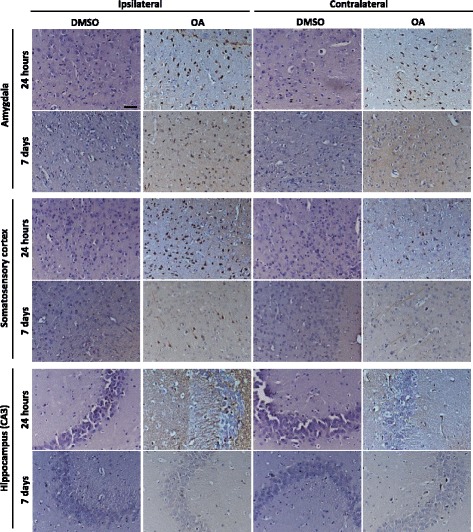
Propagation of tau phosphorylation occurs across hemispheres at 24 h at remains up to 7 days. Representative images of AT180 immunoreactivty are taken from Bregma – 1.94 at the coronal level of injection. Tau phosphorylation at AT180 is observed in both the injected and contralateral hemisphere at 24 h post OA injection. Positive neurones and neuropil staining is abundant in both the lateral amygdala and hippocampus. Furthermore positive cell bodies and processes are clearly defined in the somatosensory cortex, although qualitatively more are seen in the injected hemisphere. By 7 days phospho-tau positive neurones are predominately observed in the injected hemisphere in fewer numbers than at 24 h. Phosphorylation does not persist in the hippocampus at 7 days. Scale bar = 50 μm

**Fig. 4:**
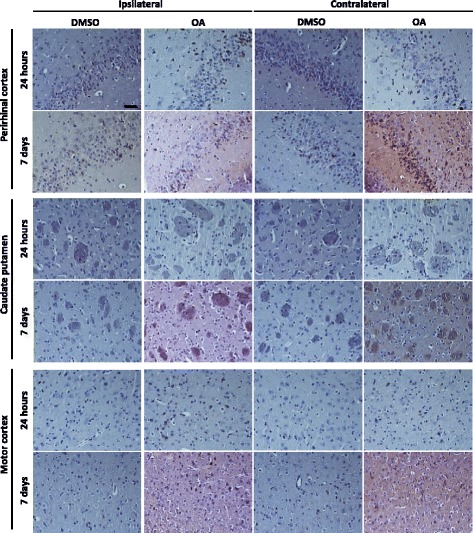
Tau phosphorylation propagates to areas anterior to the injection site. Representative images of AT180 immunoreactivty are taken from brain regions 2.08 mm anterior to the injection. At 24 h there is relatively little tau phosphorylation detectable in anterior regions, although a few positive cell bodies in the perirhinal cortex are noted. At 7 days post OA injection tau phosphorylation is pronounced in the perirhinal cortex in both the injected and contralateral hemisphere. In the striatum phospho-tau immunoreactivity is seen confined to within axon bundles. Only a small proportion of phospho-tau positive neurones are observed in the motor cortex. Scale bar = 50 μM

To characterise the tau phosphorylation pattern in response to the OA insult elicited in the lateral amygdala, we used the phospho-tau specific antibody AT180 that reacts to pathological phosphorylation at residues Thr231/Ser235 [26]. Relatively low levels of AT180 immunoreactivity were observed in DMSO-injected mice both 24 h and 7 days post-injection (Fig. 2). In the OA-injected mice, at 24 h, AT180 staining was clearly visible at the injection site of the lateral amygdala (Fig. 2b). However, phospho-tau was also abundant throughout the brain which included cortical layers and the hippocampus. Neurons showed AT180-positivity in both cell bodies and processes with substantial staining found within the neuropil, appearing in both the injected and non-injected hemisphere (Fig. 3). At 7 days post-injection, phospho-tau immunoreactivity was not as obvious in the lateral amygdala, but was instead more evident in the basal amygdala and persisted in cortical regions, predominantly the somatosensory cortex, with the most pronounced staining localised to neurons of the somatosensory cortex (Fig. 2.). However, by 7 days post-injection, AT180-positive staining in the non-injected hemisphere was greatly reduced (Fig. 3). In coronal sections taken anterior to the injection site, relatively little phospho-tau was observed at 24 h. However, at 7 days the perirhinal cortex contained both positive neurons and neuropil staining. Interestingly, although neurons within the caudate putamen were not phospho-tau-positive, AT180 immunoreactivity was observed in the axon fibre bundles passing through the region (Fig. 4).

**Fig. 5:**
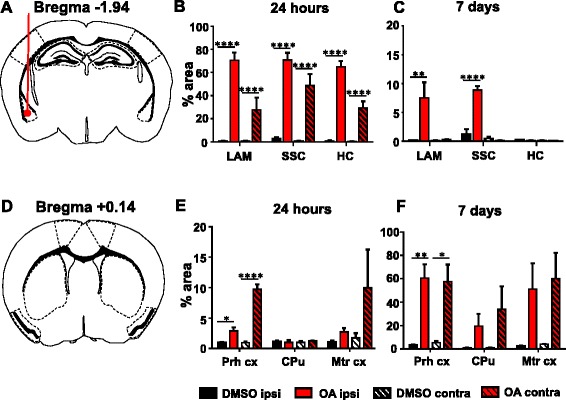
Propagation of tau pathology to anatomically distinct brain regions. Regions of interest for analysis are taken from **a** lateral amygdala (LA), somatosensory cortex (SSC) and hippocampus (HC) in sections at the level of injection and **d** perirhinal cortex (Prh cx), caudate putamen (CPu) and motor cortex (Mtr cx) from sections anterior to the injection site at Bregma + 0.14 mm. Quantification of percentage area analysis at 24 h **b**, **e** shows significant increase in phospho-tau immunoreactivity in both the ipsilateral (ipsi) and contralateral (contra) hemispheres of OA-injected mice. By 7 days significant changes are predominately noted in the ipsilateral hemisphere (**c**) with the exception of the perirhinal cortex (**f**). (*n* = 4–5, *P < 0.05, **P < 0.01, ****P < 0.0001)

To quantify the phospho-tau propagation profile induced by OA, the percentage area of phospho-tau-positive staining was determined within the brain regions of interest at the level of injection (Fig. 5a) and in sections taken anterior to the injection site (Fig. 5d). At 24 h, significant increases were recorded in the lateral amygdala, hippocampus and somatosensory cortex in both the ipsilateral and contralateral hemispheres, although significantly more phospho-tau was recorded in ipsilateral regions (Fig. 5b). When investigating regions anterior to the injection site, only the perirhinal cortex showed significant differences in phospho-tau immunoreactivity (Fig. 5e). By 7 days, no significant differences between the DMSO control and OA-injected mice were seen in the hippocampus, although significant levels of AT180-reactivity persisted in the ipsilateral lateral amygdala and somatosensory cortex, with the mean percentage area decreased from 70 % AT180-positivity to less than 10 % (Fig. 5c). Consistent with the signal observed from histology, the quantitation technique accurately demonstrated that phospho-tau is not restricted to the injection site or injection hemisphere following OA exposure.

### OA induces an increase in tau phosphorylation and insolubility over time in both hemispheres

**Fig. 6:**
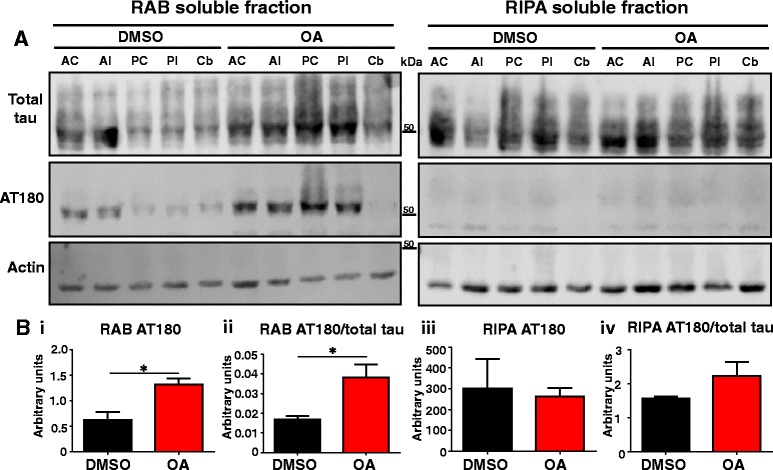
Soluble tau is hyperphosphorylated at 24 h. **a** Representative western blots for analysis of tau expression for total tau by DAKO and the phospho-tau by AT180. **b** i) Quantification using the sum of all fractions shows a significant increase in total AT180 at 24 h in OA injected mice when compared to DMSO injected controls. ii) The proportion of tau phosphorylated at AT180 is significantly increased by OA. No significant changes are observed in the insoluble, RIPA fraction (iii-iv). (*n* = 3, *p < 0.05)

To further explore whether advanced tau seeds can be formed throughout the brain in response to OA, transition of phospho-tau from a soluble to an insoluble state was explored using sequential protein extraction of dissected tissue (as for the ELISA, Fig. 1c) followed by Western blot analysis. Extraction in high-salt reassembly buffer (RAB) isolates soluble proteins and subsequent extraction in detergent-soluble radioimmunoprecipitation buffer (RIPA) isolates the remaining insoluble proteins. 24 h post-injection, there was a significant increase in tau phosphorylation at the AT180 epitope in RAB fractions obtained from the OA-injected samples compared to DMSO-injected controls (Fig. 6a, bi). Further analysis of specific fractions revealed significant phosphorylation in both the injected and non-injected hemispheres (Additional file 1: Figure S4Bi). This was reflected by an overall increase in levels of phosphorylated tau, as demonstrated by a significant increase in the ratio of AT180-phosphorylated tau to total tau (the latter determined with the DAKO antibody) (Fig. 6bii). No significant difference in total tau was observed in the RIPA-soluble fractions, and the AT180 signal was weak and unchanged at 24 h (Fig. 6biii).

**Fig. 7:**
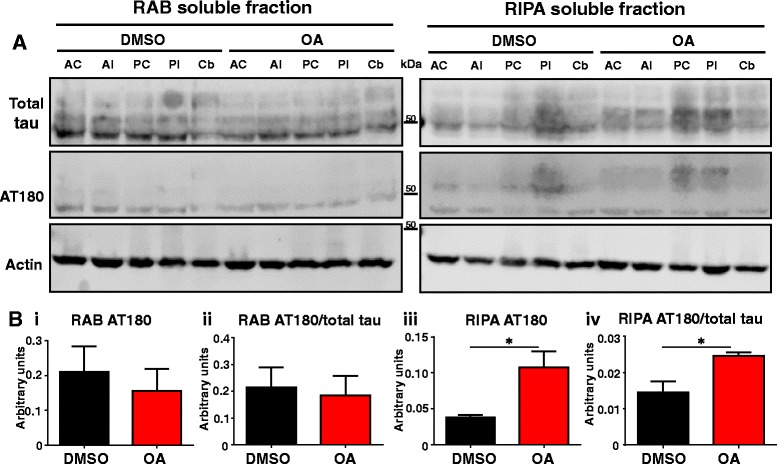
Insoluble tau is hyperphosphorylated at 7 days. **a** Representative western blots for analysis of tau expression for total tau by DAKO and the phospho-tau by AT180. **b** i) Quantification using the sum of all fractions shows no significant change in total or phospho-tau in the soluble fraction. (iii) AT180 significantly increases by 7 days in OA injected mice in the insoluble fraction compared to DMSO injected controls. The ratio of phosphorylated tau as noted by the AT180/total tau is significantly increased in the RIPA (iv) fractions but is unchanged in RAB fractions (ii). (*n* = 3, *p < 0.05)

At 7 days, no significant difference in AT180 reactivity was observed between DMSO- and OA-injected brain lysates in the RAB-soluble fraction (Fig. 7a, bi). However, AT180 phospho-tau was significantly increased in the RIPA-soluble fraction from OA-injected lysates (Fig. 7biii). An increase in molecular weight and smear in the signal was also noted, at its most pronounced in the PC and PI fractions, suggesting post-translational modifications to tau (including phosphorylation) that occurred after injection (Fig. 7a). The shift was observed across multiple phospho-tau epitopes in the PI fraction, but no significant difference was observed in nitrated-tau compared to the DMSO control, suggesting that tau hyperphosphorylation is a consistent post-translation modification contributing to the increased molecular weight (Additional file 1: Figure S5). Furthermore, the ratio of AT180 to total tau was significantly increased at 7 days. No changes in overall soluble protein content were observed in response to OA at either time-point. However, total insoluble protein content was increased in response to OA at 24 h, reflecting the observed shift in protein solubility (Additional file 1: Figure S3).

### Thioflavin staining indicates massive protein aggregation that develops rapidly and is not confined to the injection site

**Fig. 8:**
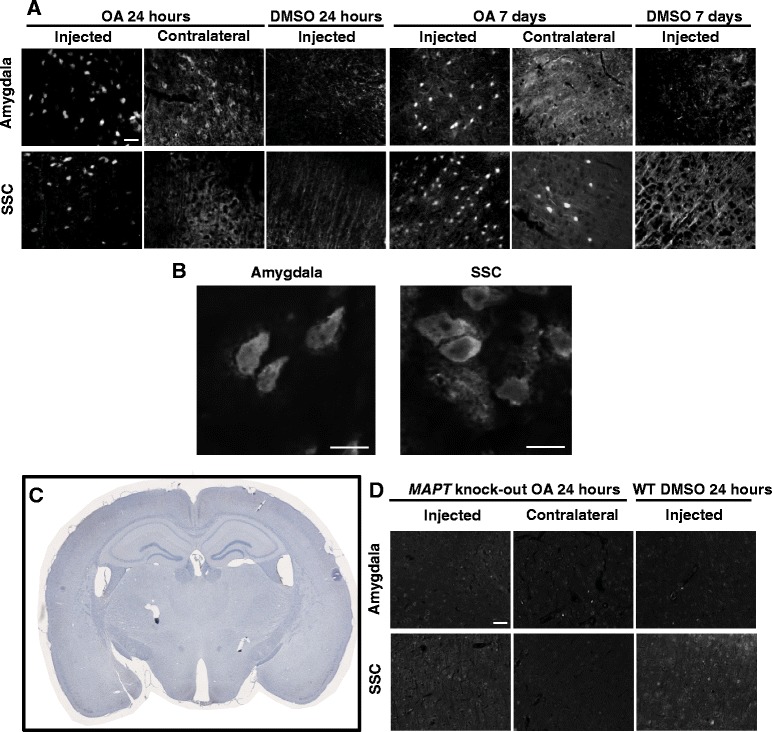
Insoluble proteins form NFT-like aggregates rapidly after OA injection at both the injection site and distal brain regions. **a** β-pleated sheet protein structures are identified by thioflavin-S. Intracellular aggregates are observed at both the injection site and distal sites in the somatosensory cortex at 24 h, which persist 7 days post injection. Scale bar = 50 μM. **b** High magnification of thioflavin-S positive cells in the amygdala and somatosensory cortex. Scale bar = 10 μm. **c** Coronal sections taken from OA injected *MAPT* KO show no phospho-tau immunoreactivity. **d** No thioflavin positive structures are observed in *MAPT* KO mice with background staining comparable to the wild-type controls at 24 h. Scale bar = 50 μm

In AD, both Aβ and tau adopt a β-pleated sheet structure as they aggregate and eventually form extracellular Aβ plaques and intracellular tau-containing NFTs, respectively. To determine whether an OA insult induces protein aggregation more generally, we used thioflavin-S, a dye with no specificity to tau, but capable of binding to β-pleated sheet structures. We found that thioflavin-positive neurons were present already after 24 h in the amygdala and also throughout the somatosensory cortex, however, they were confined to the injected hemisphere (Fig. 8a). By 7 days, thioflavin-positive neurons persisted in both the injected amygdala and the ipsilateral somatosensory cortex. In addition, a few positive neurons were also observed in the non-injected cortex (Fig. 8a). No extracellular thioflavin-S signal reminiscent of plaque staining was observed.

### Absence of protein aggregation in OA-treated tau knockout mice

To determine whether alterations to tau protein structure are integral to the formation of OA induced thioflavin-positivity, the OA injection protocol was repeated in *MAPT* knock-out mice [28], who do not express tau (Fig. 8c). Interestingly, no thioflavin-positive neurons were noted in the amygdala or the somatosensory cortex at 24 h, with background fluorescence comparable to DMSO-injected wild-type controls (Fig. 8d). Together this shows that protein aggregation elicited by OA requires the presence of tau both at the injection site and at a site distant from the injection site. This does not exclude the possibility that proteins other than tau aggregate, although in a tau-dependent manner.

## Discussion

The spreading hypothesis of tau pathology in AD in its current form originated from observations regarding the chronological order of phospho-tau immunoreactivity using phosphorylation-dependent antibodies and NFT formation in *post mortem* tissue, known as Braak staging [2]. This hypothesis dictates that template-misfolding of hyperphosphorylated tau is able to seed further protein aggregation, which can be transmitted along anatomically connected neurons, leading to a systematic and chronological progression of disease pathology. Comprehensive in vivo studies have shown that injection both of whole brain lysates and isolated recombinant tau aggregates can accelerate tau pathology at both the injection site and in neighbouring, anatomically connected brain regions when done in pre-symptomatic tau transgenic mouse models [14, 15, 17, 18]. Although this data convincingly supports the concept that pathological forms of tau can seed further aggregation of mature structural confirmation, it does not address how the initiation of primary aggregation events occur and how this triggers further dissemination throughout the neural system. Furthermore, the capacity for tau to seed and propagate aggregation in in vivo systems with endogenous tau levels remains unclear. Therefore, we used a unilateral injection of a very low volume and dose of the PP2A inhibitor OA to elicit a localised upregulation in tau phosphorylation, avoiding the introduction of preformed, mature tau seeds. In addition, wild-type mice were used to circumvent confounding interactions from over-expression of transgenic tau variants. Our results indicate that OA induces significant changes in tau phosphorylation, solubility and protein aggregation in non-exposed brain regions. Therefore, our approach could be a useful tool for further investigations into mechanisms underlying disease progression in AD.

The previous use of OA in mice involved the intracerebral injection of a tenfold higher dose of OA than used by us in a larger, 10 μl volume, in order to induce a behavioural deficit for therapeutic testing, with a focus on the oxidative stress profile rather than categorising tau phosphorylation [25]. Previous studies in rats have shown that OA induces the cell death of hippocampal CA1 neurons and the activation of heat-shock proteins in both injected and also non-injected hippocampi [23, 34]. By ELISA, we were able to detect OA in brain tissue (the first report of such a measurement in brain), which allowed us to determine the consequences of OA exposure, using DMSO injections as control. Our data provides evidence that in OA-injected mice, tau phosphorylation is increased not only in areas that were directly exposed to OA, but also in regions anterior to the injection site and in the contralateral non-injected hemisphere, where no significant OA levels were detected by ELISA. By targeting the lateral amygdala we were able to study effects on phosphorylation in brain areas significantly affected in AD as secondary sites for spreading, including the hippocampus, commonly the target in seeding experiments. Furthermore, confining OA exposure to one hemisphere allowed analysis of cross-hemispheric signalling. Interestingly, at 24 h post-injection, tau phosphorylation was significantly increased across both hemispheres, although the largest effect was observed in the injected hemisphere. Importantly, the ELISA confirmed no significant diffusion of the inhibitor between hemispheres over time, suggesting that the observed effects were due to indirect effects of OA on downstream signalling pathways to transmit tau phosphorylation. Furthermore, neighbouring brain regions displayed different responses in tau phosphorylation. For example, the perirhinal cortex had significantly increased phospho-tau immunoreactivity, whereas neighbouring striatal tissue within the same tissue showed no significant difference. Previously, the striatum has shown resistance to tau seeding when targeted directly with fibrillized recombinant tau in contrast to successful seeding in both the cortex and hippocampus [17]. Therefore, our data supports the notion that certain brain areas are more vulnerable to developing tau pathology than others, which may depend on intrinsic cell properties irrespective of connectivity, highlighting cell-autonomous mechanisms. This aspect can be further investigated by injecting OA into different brain areas to address regional differences in the ability to translate a localised PP2A inhibition into widespread alterations to tau phosphorylation.

Drastic changes in tau phosphorylation were attenuated by 7 days post injection, consistent with a decrease in detectable OA in the injected quadrant, with tau phosphorylation predominantly persisting in the injected hemisphere at much lower levels than observed at 24 h. The histology correlated well with western blotting data demonstrating remaining tau was predominantly detectable in the insoluble fraction at a higher molecular weight. This shift in solubility and size is a correlative with the progression of tau into a pathological aggregate-prone state, which typically takes months to occur in transgenic models [22] and as observed by histology, was detectable in both the injected and non-injected hemispheres. To address whether this change in solubility was reflected by protein aggregation we used the β-pleated sheet-sensitive dye thioflavin. This allowed us to detect thioflavin-positive structures at the posterior level of the injection in both the injected and non-injected hemisphere in both the cortex and amygdala at both 24 h and 7 days post-injection. Interestingly, thioflavin-reactivity was not observed in the hippocampus despite significantly elevated phospho-tau levels at 24 h, indicating that increased phospho-tau levels are not necessarily predictive of future aggregation (data not shown). In fact, transgenic human tau models present with significant levels of tau phosphorylation without developing end-stage thioflavin or Gallyas-positive lesions [31, 32], unless the mice become very old [35]. Qualitatively, these thioflavin-positive aggregates appear to increase in numbers over time, suggesting that while phosphorylation is dynamic and transient, once proteins adopt an aggregated state these forms tend to persist. Importantly, the presence of tau is crucial to the formation of thioflavin-positivity as evidenced by the lack of staining in OA-injected tau knock-out controls. Although the mechanism by which tauopathy crosses hemispheres in this model requires further investigation, our data presents evidence that effects of tau spreading can be observed over short-time frames, without the need for human transgene expression or injection of preformed seeds.

A limitation of our study is that the tau we observe is unlabelled, thus we are unable to track specific tau species as they propagate from neuron to neuron. This could be addressed in subsequent studies by using gene editing methods to generate a mouse strain that expresses tagged forms of endogenous tau with a restricted expression pattern, however, this would again introduce incomplete restriction of transgene expression as a possible confound. Currently, it is not known whether the phospho-tau species observed in our study are a result of a direct spreading of tau originating in the lateral amygdala, or a result of the communication of a stress signal translated into phosphorylation. A plausible explanation given the short time frame in which tau phosphorylation disperses throughout the brain is that the accumulation of pathological tau provides a metabolic insult within a neuron which causes downstream dysfunction through trans-synaptic signalling. However, one can assume that it is likely that both direct tau spreading and metabolic injury act concurrently in disease progression, both in our experimental paradigm and in a human disease setting. Approaches that utilise OA offer the potential to further explore this hypothesis.

## Conclusions

In summary, the data presented here suggests that localised OA injections may be a viable avenue for better understanding the initiation and cell vulnerability underlying the spreading of tau. Our findings strongly suggest that tau phosphorylation can propagate rapidly across hemispheres following a confined unilateral insult, resulting in significant changes in tau solubility and tau-dependent protein aggregation. Moreover, these responses can be observed over short time frames in wild-type mice that only express endogenous tau. Future studies to better understand the underlying mechanisms are warranted, while application of OA to different brains regions may assist in clarifying cell-autonomous mechanisms necessary for the initiation of tau spreading.

## Additional file

Additional file 1: Figure S1. Detection sensitivity of the OA ELISA. A) In water, OA is detectable down to spiked concentrations of 0.075 ng, with little background signal in the water only control. B) As positive control, homogenized brain was spiked with 100 ng OA, a 10x higher dose than used experimentally. OA is detected at significant levels in positively spiked tissue compared to non-spiked brain homogenate. A slight background signal is detected in the non-spiked samples suggesting low levels of non-specific binding in brain tissue compared to water (*n* = 3, ***p* < 0.01). **Figure S2.** Total protein content assessed by the sum of protein concentration from all fractions per animal reveals no significant difference between RAB fractions. At 24 h, OA induces a small but significant increase in the insoluble RIPA fraction which persists, but is no longer significant by 7 days (*n* = 3, **p* < 0.05). **Figure S3.** Western blotting quantification at 24 h assessing A,D) soluble total tau, B,E) AT180-phosphorylated tau, and C,F) the phospho-tau/total tau ratio. **Figure S4.** Quantification of fractions at 7 days, observing no significant differences in trend or mean for the RAB fractions (A,B,C). D) Insoluble total tau levels are non-significantly elevated by OA in the posterior quadrants. E) OA-treatment significantly increases AT180 phosphorylation in both injected and contralateral posterior quadrants. F) Overall trend for increased phospho/total tau ratio in the soluble fractions for all quadrants (*n* = 3, **p* < 0.05). **Figure S5.** Multiple tau residues are hyperphosphorylated in the insoluble fraction at 7 days. Representative blots from RIPA fractions probed for total tau (DAKO) and phosphospecific antibodies A) AT8, B) S422, C) AT270, D) S235, and E) S262. All phospho-antibodies show the most prominent increase in phospho-tau in the OA-injected PI fraction (see also the additional higher molecular weight signal). F) No significant differences were detected with a nitrated tau-specific antibody. (PDF 1020 kb)

## Abbreviations

AD: Alzheimer’s disease
NFT: neurofibrillary tangle
OA: okadaic acid
PP2A: protein phosphatase 2A
RAB buffer: high-salt reassembly buffer
RIPA buffer: radio-immunoprecipitation buffer
