# Supplementary material for: A local insult of okadaic acid in wild-type mice induces tau phosphorylation and protein aggregation in anatomically distinct brain regions
Source: Acta Neuropathol Commun. 2016 Mar 31;4:32. doi: 10.1186/s40478-016-0300-0 (PMC4818468; doi:10.1186/s40478-016-0300-0)

Supplementary figure 1

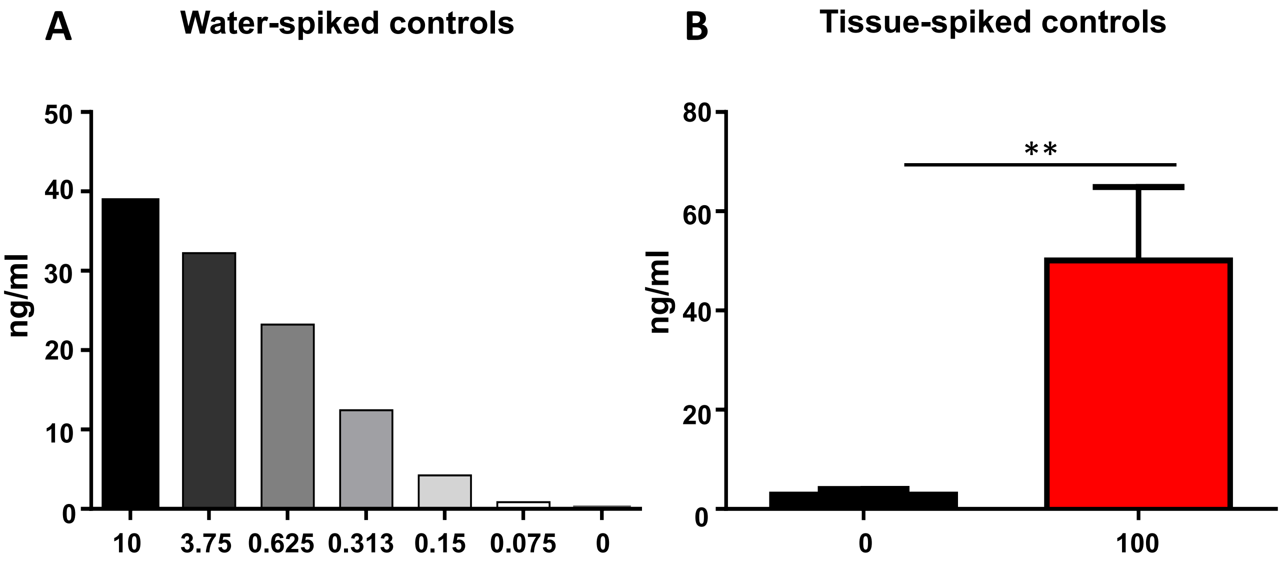

Supplementary figure 2

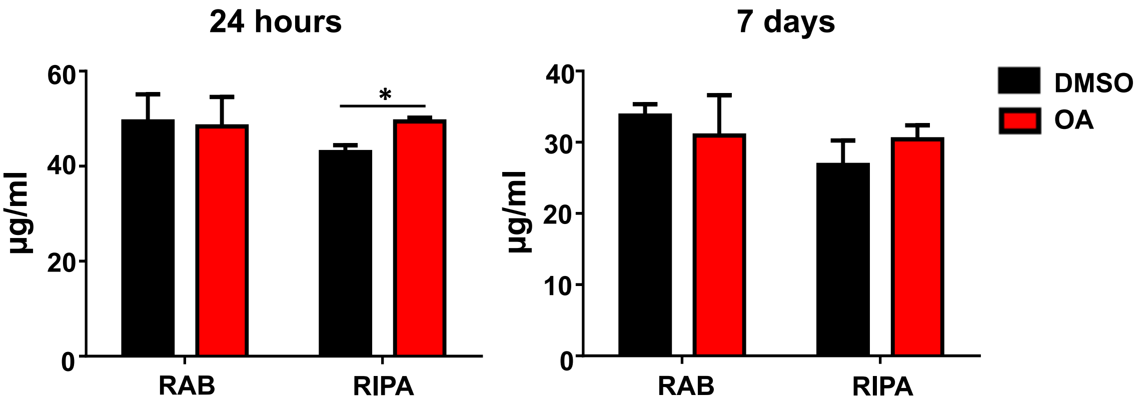

Supplementary figure 3

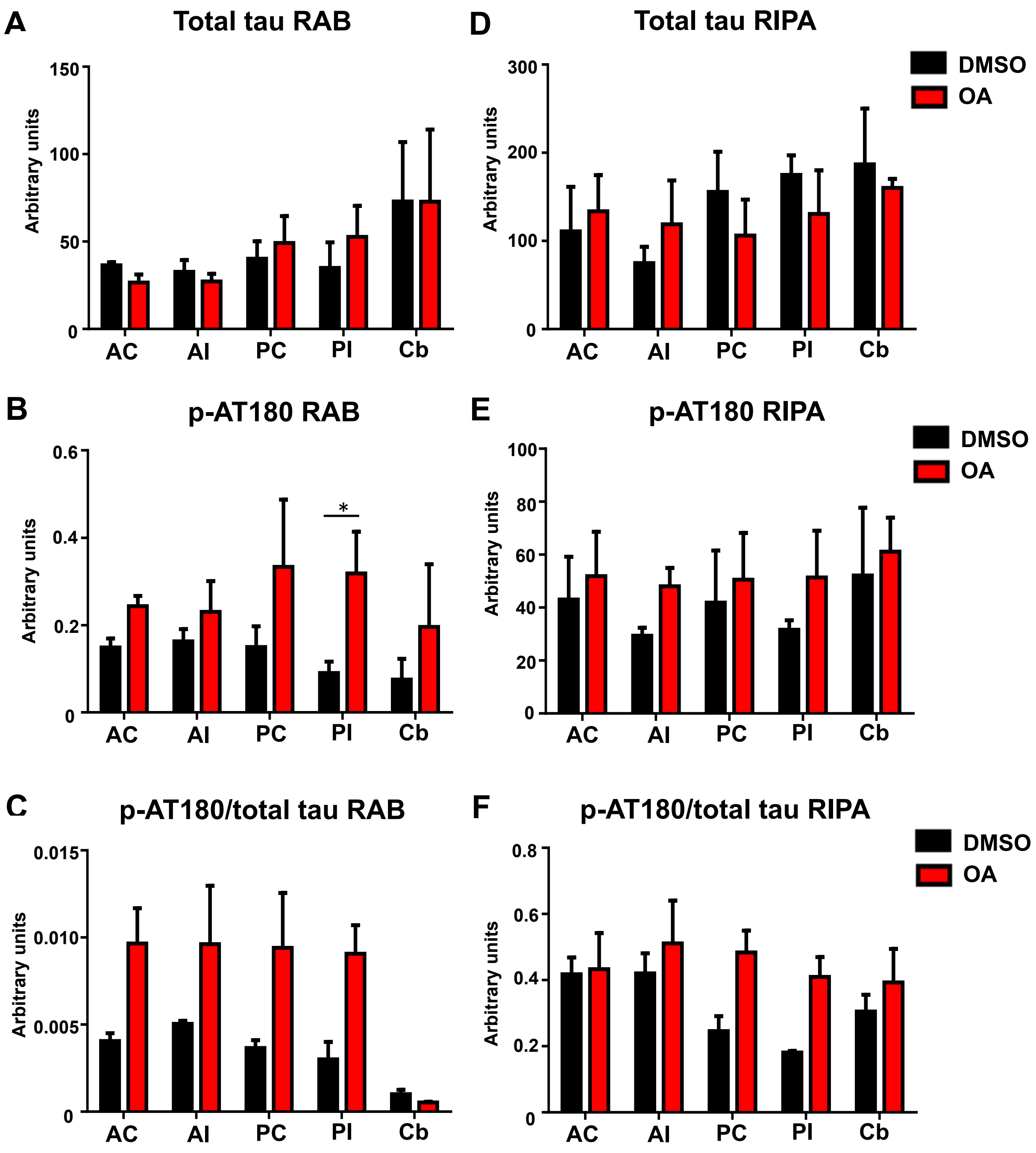

Supplementary figure 4

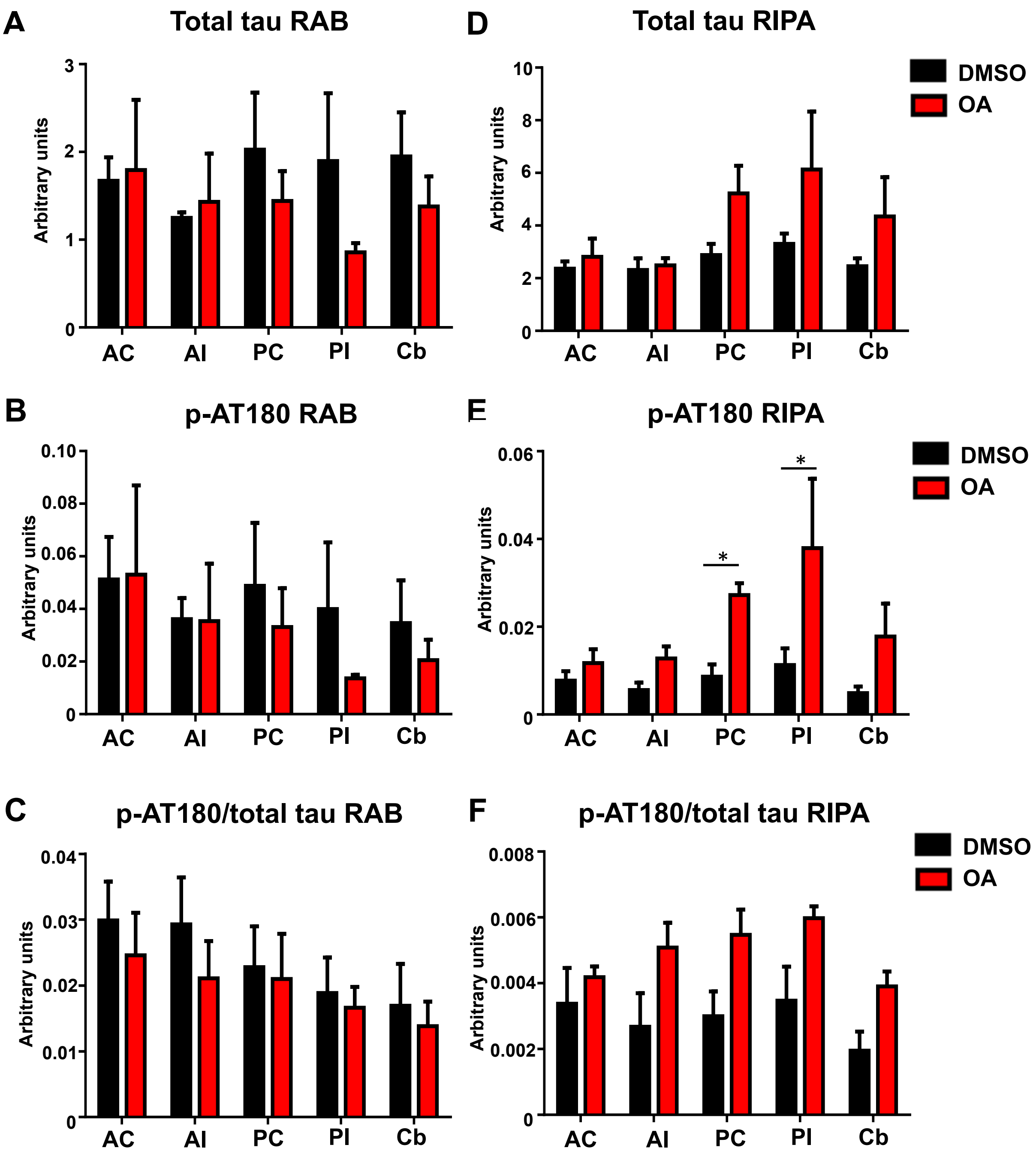

Supplementary figure 5

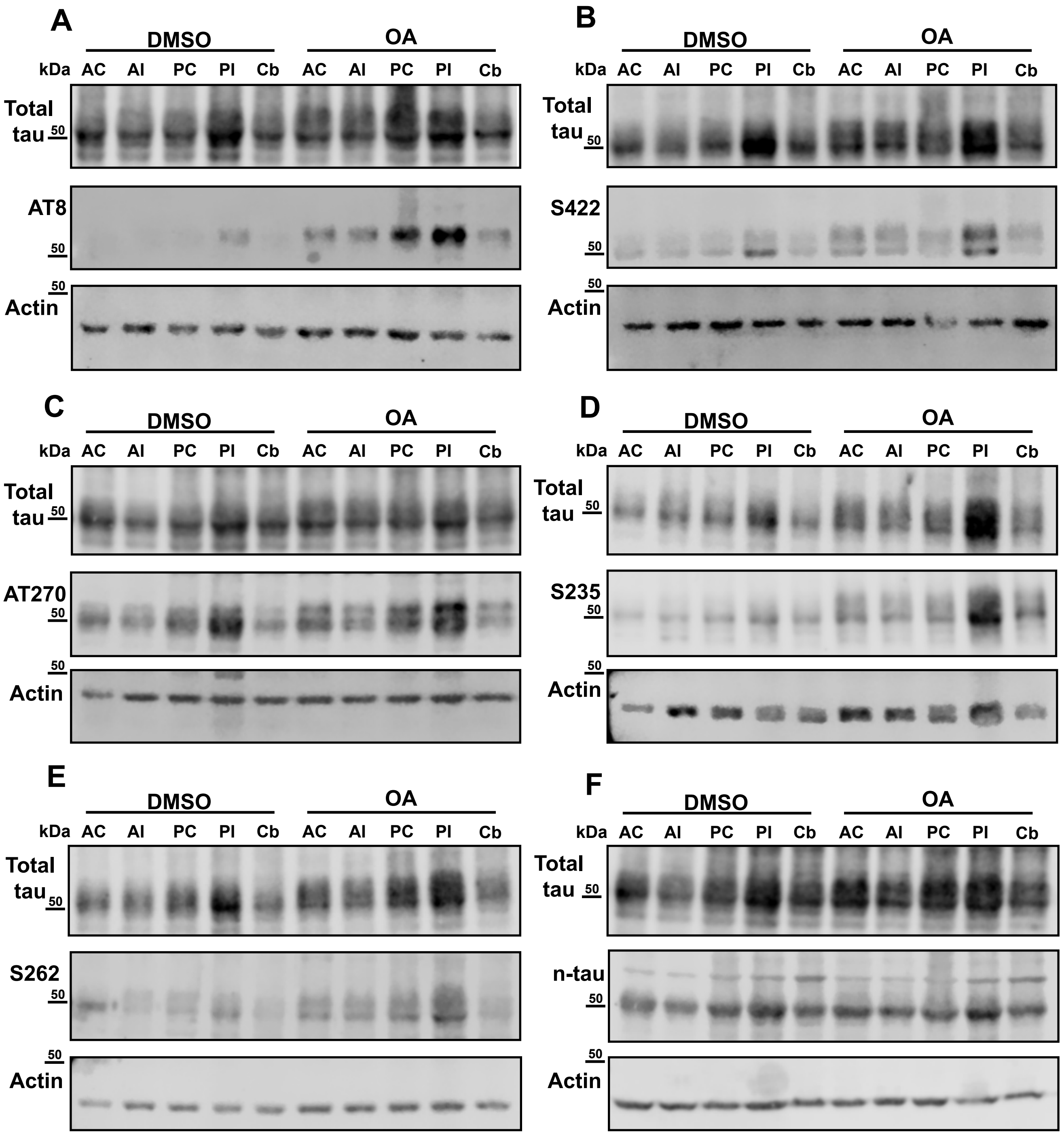

Supplement: Additional file 1: Figure S1. — Detection sensitivity of the OA ELISA. A) In water, OA is detectable down to spiked concentrations of 0.075 ng, with little background signal in the water only control. B) As positive control, homogenized brain was spiked with 100 ng OA, a 10x higher dose than used experimentally. OA is detected at significant levels in positively spiked tissue compared to non-spiked brain homogenate. A slight background signal is detected in the non-spiked samples suggesting low levels of non-specific binding in brain tissue compared to water (n = 3, **p < 0.01). Figure S2. Total protein content assessed by the sum of protein concentration from all fractions per animal reveals no significant difference between RAB fractions. At 24 h, OA induces a small but significant increase in the insoluble RIPA fraction which persists, but is no longer significant by 7 days (n = 3, *p < 0.05). Figure S3. Western blotting quantification at 24 h assessing A,D) soluble total tau, B,E) AT180-phosphorylated tau, and C,F) the phospho-tau/total tau ratio. Figure S4. Quantification of fractions at 7 days, observing no significant differences in trend or mean for the RAB fractions (A,B,C). D) Insoluble total tau levels are non-significantly elevated by OA in the posterior quadrants. E) OA-treatment significantly increases AT180 phosphorylation in both injected and contralateral posterior quadrants. F) Overall trend for increased phospho/total tau ratio in the soluble fractions for all quadrants (n = 3, *p < 0.05). Figure S5. Multiple tau residues are hyperphosphorylated in the insoluble fraction at 7 days. Representative blots from RIPA fractions probed for total tau (DAKO) and phosphospecific antibodies A) AT8, B) S422, C) AT270, D) S235, and E) S262. All phospho-antibodies show the most prominent increase in phospho-tau in the OA-injected PI fraction (see also the additional higher molecular weight signal). F) No significant differences were detected with a nitrated tau-specific antibody. (PDF 1020 kb [file 40478_2016_300_MOESM1_ESM.pdf]
